# Anti-Methicillin-Resistant *Staphylococcus aureu*s Activities of *Artocarpus lakoocha* Roxb Extract and Its Mode of Action

**DOI:** 10.1155/2022/1839356

**Published:** 2022-06-15

**Authors:** Punyisa Charirak, Khakhanang Ratananikom

**Affiliations:** ^1^Department of Plant Production Technology, Faculty of Agricultural Technology, Kalasin University, Kalasin, Thailand; ^2^Department of Science and Mathematics, Faculty of Science and Health Technology, Kalasin University, Kalasin, Thailand

## Abstract

Antibacterial activities and mode of action of *Artocarpus lakoocha* Roxb extracts against methicillin-resistant *Staphylococcus aureus* (MRSA) were evaluated, with its biological properties including antioxidant activity and total phenolic content also measured. Heartwoods of *A. lakoocha* Roxb were extracted by solvents including water, ethanol, acetonitrile, ethyl acetate, isopropanol, and hexane. Results showed that antibacterial activity against MRSA, antioxidant activity, and total phenolic content were highest in the acetonitrile extract. Minimum inhibitory concentration (MIC) and minimum bactericidal concentration (MBC) were 312.5 and 625.0 *μ*g/mL, respectively. Time-killing evaluation indicated a bactericidal mode of action with IC_50_ values against ABTS and DPPH radicals of 2.37 ± 0.09 and 32.10 ± 0.74 mg/mL, respectively, and total phenolic content 455.29 ± 18.35 mg GAE/g extract. Results suggested that acetonitrile extract of *A. lakoocha* Roxb had good potential activity against MRSA, with promise for further development as a novel alternative drug.

## 1. Introduction

Drug-resistant bacteria are a serious problem worldwide. Some bacteria have developed the ability to survive in the presence of drugs that were designed to kill them [1, 2]. People with drug-resistant bacterial infections require longer stay in hospital and higher expenditure from extended medical treatment, with greater risk of death at approximately 3.5% [3]. The most common drug-resistant bacterium is methicillin-resistant *Staphylococcus aureus* (MRSA) that causes high rates of morbidity and mortality [4]. In the United States, about 20,000 out of 120,000 patients infected by MRSA died in 2017 [5]. Overuse and/or misuse of antibiotics [1], poor sanitation [6], poverty [7], and international travel [8] are all causes of drug-resistant bacteria, now recognized as a high-priority threat to human health. To overcome this problem, demand for screening newer and alternative compounds from nature, especially plants, has mushroomed [1, 2].

Plants have long been recognized as valuable sources of primary human health care offering a vast diversity of secondary metabolites including alkaloids, anthraquinones, carotenoids, flavonoids, tannins, lignin, and phenolic compounds with relatively smaller quantities than primary metabolites [1]. These bioactive compounds have pharmacological properties and also demonstrate antimicrobial activities [9–13]. Plant-derived compounds offer safer, more effective, and multifunctional treatment with fewer side effects than synthetic drugs [1, 12].

*Artocarpus lakoocha* Roxb is a perennial tree belonging to the Moraceae family. This traditional herbal medicine is found in tropical areas such as Thailand, Malaysia, and India [14]. It is called lokhat in Thailand, tampang in Malaysia, and lakuchi in India. This tree is used as a traditional medicine due to the availability of abundant bioactive compounds in its bark, leaves, seeds, fruit, and heartwood. These bioactive compounds display therapeutic activities [14]. For example, oxyresveratrol, a major constituent in the heartwood revealed anti-herpes simplex virus, anthelmintic, antimicrobial, and antibiofilm activity [15, 16], while squalene, 3,4-dihydroxymandekic acid, 9-octyl eicosane, and 7,8-didehydro-3-methyoxy-17-methyl-6-methylene morphinan which are mostly found in *Artocarpus* leaves showed antimicrobial and antioxidant activities [17, 18]. Moreover, *A. lakoocha* extract showed antityrosinase activity and is used as a whitening agent in cosmetics [19]. *A. lakoocha* Roxb has proven antimicrobial activity against several pathogenic bacteria but knowledge of its inhibitory effect on drug-resistant bacteria remains limited. Therefore, here, we (1) evaluated anti-MRSA activity of *A. lakoocha* Roxb extracts, (2) investigated the mode of action of *A. lakoocha* Roxb extracts against MRSA, and (3) examined the antioxidant activity and total phenolic contents of the extracts.

## 2. Materials and Methods

### 2.1. Materials

Methicillin-resistant *Staphylococcus aureus* ATTC 43300 was used as the indicator strain. Nutrient broth and agar powder at bacteriological grade were purchased from HiMedia, India. Ethyl acetate, isopropanol, and hexane at AR grade were purchased from KemAus, Australia, while ethanol and acetonitrile at AR grade were purchased from Merck, Germany.

### 2.2. Preparation of *A. lakoocha* Roxb Extracts

Dried powder of heartwoods from *A. lakoocha* Roxb was purchased from a Thai traditional drug store in Bangkok, Thailand. The powder was extracted by solvents with different polarity including water, ethanol, acetonitrile, ethyl acetate, isopropanol, and hexane at a ratio of 1 : 10 (powder : solvent) via the maceration technique for 24 hours in an incubator shaker at 150 rpm. The solutions were filtrated through Whatman filter papers and concentrated by a rotary evaporator. The extracts were weighed and stored in airtight bottles at 4°C for further study.

### 2.3. Antibacterial Screening

Agar-disc diffusion was used to screen for the antibacterial activity of *A. lakoocha* Roxb extracts against MRSA. Briefly, MRSA was grown in nutrient broth at 30°C for 16–18 hours to obtain a bacterial concentration of 10^8^ CFU/mL. Sterile discs (6 mm diameter) were placed on solidified nutrient agar, covered with 100 *μ*L of MRSA. Ten microliters of *A. lakoocha* Roxb extracts were dropped onto the sterile discs and the nutrient agar plates were incubated at 30°C for 24 hours. Antibacterial activity was determined by measuring the inhibition zone diameter (mm). All experiments were conducted in triplicate.

### 2.4. Determination of Minimum Inhibitory Concentration and Minimum Bactericidal Concentration

Minimum inhibitory concentration (MIC) and minimum bacterial concentration (MBC) were determined using the resazurin microtiter assay with some modifications [20]. One hundred microliters of nutrient broth were pipetted into a 96-well plate. A two-fold serial dilution technique was carried out to prepare various concentrations of the *A. lakoocha* Roxb extracts. One hundred microliters of MRSA were added into the mixtures. The 96-well plates were incubated at 3 C for 16–18 hours. After that, 30 *μ*L of 0.02% resazurin was added into each well and the 96-well plates were further incubated at 30°C for 16–18 hours to obtain the MIC. The MIC was observed as no color change of resazurin, indicating the lowest concentration of *A. lakoocha* Roxb extracts with inhibitory property against the indicator strain. Then, 100 *μ*L of the mixture in each well was plated on solidified nutrient agar and incubated at 30°C for 24 hours to obtain the MBC. The MBC was observed by no growth on nutrient agar, indicating that 95% inoculation was killed. The MBC was defined as the lowest concentration of *A. lakoocha* Roxb extract with bactericidal activity against the indicator strain. All experiments were conducted in triplicate.

### 2.5. Time-Killing Assay

The time-killing curve of the acetonitrile extract from *A. lakoocha* Roxb was carried out by following the procedure of Appiah et al. with some modifications [9]. The acetonitrile extract of *A. lakoocha* Roxb at twice the MIC was prepared and then the MRSA culture at a concentration of 10^6^ CFU/mL was added. The mixture was incubated at 30°C in an incubator shaker at 150 rpm. An aliquot of 100 *μ*L of the medium was removed at time intervals of 0, 1, 2, 3, 4, 5, 6, 7, and 8 hours after innoculation. Growing cells of MRSA in nutrient broth without *A. lakoocha* Roxb extract were used as the control. The number of visible cells was recorded by plating on nutrient agar. All procedures were performed in triplicate and a graph of log_10_ (CFU/mL) was plotted against time.

### 2.6. Free Radical Scavenging Test by ABTS Radicals

Free radical scavenging activity of *A. lakoocha* Roxb extracts was examined by the ABTS radical cation decolorization method with some modifications [21]. The ABTS cation radicals were prepared using a mixture of 2.45 mM potassium persulfate and 7 mM ABTS in distilled water at a ratio of 1 : 1. The mixture was incubated in darkness at room temperature for 16 hours before use. Various concentrations of *A. lakoocha* Roxb extracts and ABTS cation radicals were mixed and incubated in darkness at room temperature for 30 min. Absorption was measured at a wavelength of 734 nm. The antioxidant effectiveness of *A. lakoocha* Roxb extracts was calculated using the formula:

(1) $$\begin{array}{c} \text{ABTS scavenging effect}\left(\%\right)=\left[\frac{\left({\text{Abs}}_{\text{B}}{-\text{Abs}}_{\text{A}}\right)}{{\text{Abs}}_{\text{B}}}\right]\times 100, \end{array}$$

where Abs_B_ and Abs_A_ are the absorbances of the mixture without *A. lakoocha* Roxb extract and with *A. lakoocha* Roxb extract, respectively. Linear regression of antioxidant effectiveness was used to determine the concentration of *A. lakoocha* Roxb extract that could reduce the concentration of ABTS radicals by 50% (IC_50_).

### 2.7. Free Radical Scavenging Test by DPPH Radicals

Free radical scavenging activity of *A. lakoocha* Roxb extracts was examined by the DPPH radical method, with some modifications [22]. Briefly, 0.1 mM DPPH radicals were prepared in ethanol, mixed with various concentrations of *A. lakoocha* Roxb extracts, and then incubated in darkness at room temperature for 30 min. Absorption was measured at a wavelength of 517 nm. The antioxidant effectiveness of *A. lakoocha* Roxb extracts was calculated using the formula:

(2) $$\begin{array}{c} \text{DPPH scavenging effect}\left(\%\right)=\left[\frac{\left({\text{Abs}}_{\text{B}}{-\text{Abs}}_{\text{A}}\right)}{{\text{Abs}}_{\text{B}}}\right]\times 100, \end{array}$$

where Abs_B_ and Abs_A_ are the absorbances of the mixture without *A. lakoocha* Roxb extract and with *A. lakoocha* Roxb extract, respectively. Linear regression of antioxidant effectiveness was used to determine the concentration of *A. lakoocha* Roxb extract that could reduce the concentration of DPPH radicals by 50% (IC5_0_).

### 2.8. Determination of Total Phenolic Content

Total phenolic contents were determined by the Folin–Ciocalteu method with some modifications [23]. First, 0.5 mL of *A. lakoocha* Roxb extract was mixed with 10% Folin–Ciocalteu reagent and incubated at room temperature for 10 min. Then, 7.5% sodium carbonate was added and the mixture was incubated for 60 min at room temperature. Absorption was evaluated at a wavelength of 765 nm. Total phenolic content was determined by comparison with the gallic acid standard curve and expressed as mg gallic acid equivalent (GAE)/g extract.

### 2.9. Statistical Analysis

Statistix version 8.0 was used for statistical analysis. Data were expressed as mean ± standard deviation. Measurements with normal distribution were analyzed using one-way analysis of variance (ANOVA), followed by the least significant difference (LSD) with a significance level of *α* = 0.05.

## 3. Results

### 3.1. Preparation of *A. lakoocha* Roxb Extracts

Yields of *A. lakoocha* Roxb extracts were different according to type of extraction solvent used. Highest yield was recorded in the water extract, with the lowest as the hexane extract with a 5-fold difference (Table 1).

### 3.2. Antibacterial Screening

**Figure 1:**
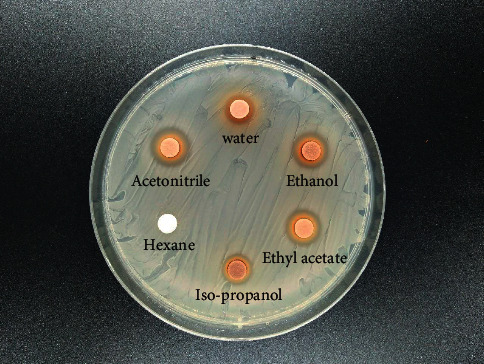
Anti-MRSA screening of *A. lakoocha* roxb extracts.

Figure 1 and Table 2 show the antibacterial screening results. Anti-MRSA activities were not found in hexane, while the other extracts, with acetonitrile showing the largest inhibition zone diameter (12.41 ± 0.06 mm) followed by ethyl acetate, ethanol, isopropanol, and water extracts at 11.91 ± 0.11, 11.06 ± 0.12, 9.77 ± 0.14, and 8.74 ± 0.20 mm, respectively.

### 3.3. MIC and MBC Determination

The MICs and MBCs of *A. lakoocha* Roxb extracts against MRSA are summarized in Table 3. Results corresponded with those from antibacterial screening by agar-disc diffusion. The *A. lakoocha* Roxb extracts showed different MICs and MBCs. The acetonitrile extract exhibited highest activity against MRSA with lowest MIC and MBC values at 312.5 and 625.0 *μ*g/mL, respectively. By contrast, MRSA growth was not affected by the highest concentration of the hexane extract, which clearly indicated no anti-MRSA activity.

### 3.4. Mode of Action of *A. lakoocha* Roxb Extracts against MRSA

**Figure 2:**
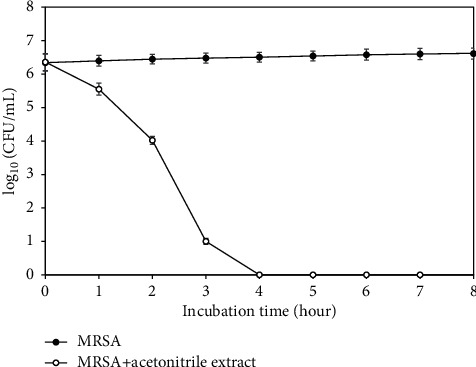
Time-killing curve of the acetonitrile extract of *A lakoocha* roxb against MRSA.

The acetonitrile extract of *A. lakoocha* Roxb with the lowest MIC and MBC values, indicating highest MRSA inhibitory activity, was selected to study its mode of action. Cell viability of MRSA grew continuously over time, while a dramatic reduction in cell viability was found when MRSA was treated with the acetonitrile extract. MRSA was completely destroyed within 4 hours after incubation by the acetonitrile extract (Figure 2).

### 3.5. Antioxidant Activity and Total Phenolic Content of *A. lakoocha* Roxb Extracts

Table 4 shows the scavenging activities of *A. lakoocha* Roxb extracts and their total phenolic contents. The acetonitrile extract showed the best scavenging activity against ABTS and DPPH radicals with lowest IC_50_ values at 2.37 ± 0.09 and 32.10 ± 0.74 mg/mL, respectively. Total phenolic content of the acetonitrile extract was also highest at 455.29 ± 18.35 mg GAE/g extract. Other extracts had different antioxidant activities and total phenolic contents, with the hexane extract exhibiting lowest antioxidant activity and total phenolic content.

## 4. Discussion

Results revealed that the type of solvent extraction played an important role on the isolation of bioactive compounds from *A. lakoocha* Roxb. Acetonitrile, as the optimal extraction solvent, gave the highest antibacterial activity, antioxidant activity, and total phenolic content. This finding was supported by Shanmughapriya et al. and Lima-Filho et al. who explained that the type of extraction solvent impacted the biological activities and plant-deviated constituents in crude extracts. Organic solvents usually demonstrated higher efficiency in extracting active compounds for antimicrobial activities compared with water-based methods [24, 25].

Results of antibacterial screening by agar-disc diffusion and MIC and MBC values by the broth dilution assay concurred well, indicating the potential anti-MRSA activity of the acetonitrile extract to diffuse into agar and distinguish bactericidal and bacteriostatic effects. Modes of action for antimicrobial agents are defined as bactericidal or bacteriostatic, based on the ratio of MBC/MIC. When the MBC/MIC ratio is ≤4, this is defined as bactericidal with bacteriostatic effect at >4 [9, 26]. In this study, the ratios obtained from *A. lakoocha* Roxb extracts, apart from the acetonitrile extract, were about 4 but the MBC/MIC ratio of the acetonitrile extract was interestingly lower than 4. This finding indicated the bactericidal effect of the acetonitrile extract of *A. lakoocha* Roxb against MRSA. The mode of action as bactericidal of the acetonitrile extract of *A. lakoocha* Roxb against MRSA was also confirmed by the time-killing assay. Within 4 hours after inoculation, MRSA growth was completely destroyed, strongly indicating that the acetonitrile extract of *A. lakoocha* Roxb exhibited bactericidal action against MRSA.

The acetonitrile extract of *A. lakoocha* Roxb exhibited anti-MRSA activity and also demonstrated potential as a good source of antioxidants and phenolic compounds. The antibacterial agents, antioxidants, and phenolic compounds available in the acetonitrile extract of *A. lakoocha* Roxb tended to have hydrophilic properties and preferred to dissolve in polar solvents when compared with hexane. This result was supported by several studies indicating that *A. lakoocha* Roxb was a very good source of antimicrobial agents, antioxidants, and phenolic compounds. Common extraction solvents used to obtain extracts with antimicrobial and antioxidant activities and phenolic content were ethanol, methanol, ethyl acetate, and acetonitrile [10, 14–19].

The significant anti-MRSA property and antioxidant activity of *A. lakoocha* Roxb occurred as a result of the synergistic manner of active components available in the acetonitrile extract. This was an advantage of using crude extract that battled pathogenic bacteria. Due to several active components, bacteria cannot easily resist against a combination of active compounds. By contrast, synthetic antibiotic usually presents antibacterial properties by causing the bacteria to develop resistant mechanisms. This explanation was supported by results from the ethanolic extract of cinnamon bark and honey [12]. The combination of cinnamon bark and honey showed a synergistic effect against acne-causing bacteria over the individual ethanolic extracts of cinnamon bark and honey. The active compounds found in the acetonitrile extract could possibly combat MRSA via several mechanisms which previously described, including efflux pump inhibition, bacteria membrane destruction, biofilm inhibition, and DNA and protein retardation [1]. They also collaboratively provided electrons or hydrogen to scavenge free radicals [1]. The degree of antibacterial activity depended on the type and dose of the active compounds synthesized in plants. Plants produce primary metabolites for growth and survival as well as secondary metabolites that adapt to the environment to combat injury, temperature, moisture, and stress. These secondary metabolites including alkaloids, polyphenols, terpenoids, and sulfur-containing compounds have been previously reported as having antimicrobial activity via different mechanisms. Several factors such as geological area, harvesting season, climate, and plant part also contribute to the antibacterial activity of plant extracts [1].

## 5. Conclusions

Our investigations focused on evaluating the *in vitro* inhibitory activities of *A. lakoocha* Roxb extracts against MRSA. The acetonitrile extract showed significant anti-MRSA activity and highest antioxidant activity and total phenolic content with MIC and MBC values of 312.5 and 625.0 *μ*g/mL, respectively. Its mode of action was also defined as bactericidal. Results suggested that the acetonitrile extract of *A. lakoocha* Roxb showed high potential inhibitory activity against MRSA and could be further developed as a novel alternative drug.

## Figures and Tables

**Table 1:** Yields of *A. lakoocha* roxb extracts.

Extraction solvent	Percent yield (%)
Water	4.23
Ethanol	3.25
Acetonitrile	3.02
Ethyl acetate	3.50
Isopropanol	2.90
Hexane	0.75

**Table 2:** Inhibition zone diameters of *A. lakoocha* roxb extracts against MRSA.

Extraction solvent	Inhibition zone diameter (mean ± SD, mm)
Water	8.74 ± 0.20^e^
Ethanol	11.06 ± 0.12^c^
Acetonitrile	12.41 ± 0.06^a^
Ethyl acetate	11.91 ± 0.11^b^
Isopropanol	9.77 ± 0.14^d^
Hexane	6.00 ± 0.00^f^

Different letters in the column indicate significant difference (*P* < 0.01).

**Table 3:** MIC and MBC of *A. lakoocha* roxb extracts against MRSA.

Extraction solvent	MIC (*μ*g/mL)	MBC (*μ*g/mL)	MBC/MIC ratio
Water	1,250	5,000.0	4
Ethanol	625.0	2,500.0	4
Acetonitrile	312.5	625.0	2
Ethyl acetate	312.5	1,250.0	4
Isopropanol	625.0	2,500.0	4
Hexane	>5,000	>5,000	nd

**Table 4:** Antioxidant activity and total phenolic content of *A. lakoocha* roxb extracts.

Extraction solvent	ABTS IC_50_ (mg/mL)	DPPH IC_50_ (mg/mL)	Total phenolic content (mg GAE/g extract)
Water	3.05 ± 0.04^b^	47.90 ± 2.05^b^	318.42 ± 4.72^d^
Ethanol	2.40 ± 0.08^b^	36.88 ± 1.62^bc^	423.53 ± 11.48^b^
Acetonitrile	2.37 ± 0.09^b^	32.10 ± 0.74^c^	455.29 ± 18.35^a^
Ethyl acetate	2.40 ± 0.07^b^	36.32 ± 1.43^bc^	416.47 ± 5.97^b^
Isopropanol	2.82 ± 0.15^b^	39.25 ± 1.92^bc^	372.35 ± 4.44^c^
Hexane	5,005.34 ± 200.34^a^	449.68 ± 19.82^a^	33.33 ± 0.34^e^

Different letters in the columns indicate significant difference (*P* < 0.01).

## Data Availability

The data on antibacterial screening, determination of MIC and MBC, time-killing assay, and determination of antioxidant activity used to support the findings of this study are available from the corresponding author upon request.
